# Brain iron distribution in transdiagnostic mental health burden

**DOI:** 10.1002/pcn5.70243

**Published:** 2025-11-19

**Authors:** Rebecca Christina Coray, Jatta Berberat, Sonja Maria Kagerer, Nader Perroud, Lopez Julian Gaviria, Camille Piguet, Paul Gerson Unschuld

**Affiliations:** ^1^ Department of Psychiatry University of Geneva (UniGE) Geneva Switzerland; ^2^ Institute of Neuroradiology, Kantonsspital Aarau Aarau Switzerland; ^3^ Geriatric Psychiatry and Psychotherapy University Hospital of Psychiatry Zurich (PUK) Zurich Switzerland; ^4^ Department of Psychiatry Vrije Universiteit Amsterdam The Netherlands; ^5^ Geriatric Psychiatry Service University Hospitals of Geneva (HUG) Thônex Switzerland

**Keywords:** brain iron, hierarchical clustering, iron homeostasis, multi‐parameter mapping, psychiatric disorders, transdiagnostic biomarkers

## Abstract

**Background:**

Psychiatric diseases are increasingly understood as a spectrum or continuous phenomena, ranging from healthy to severely affected, yet neurobiological correlates for these dimensions remain elusive. Regional alteration of iron levels in the central nervous system may reflect neuropathological processes that result in significant impairment of cognitive and behavioral functions. The aim of this study was to characterize brain iron distribution in individuals with common clinical psychiatric disorders and offspring of individuals affected by these disorders.

**Methods:**

R2* magnetic resonance imaging (MRI) based multi‐parameter mapping (MPM) algorithm was used to assess regional brain iron distribution in a) individuals diagnosed with bipolar disorder (BD), borderline personality disorder (BPD), attention‐deficit/hyperactivity disorder (ADHD), and offspring (*n* = 80, age = 23 ± 7 y., 61% females), and b) healthy controls and offspring controls (*n* = 43, age = 25 ± 9, 56% females). Hierarchical cluster analysis (HCA) was used to identify group‐divisive patterns of regional brain iron distribution.

**Results:**

Three distinct clusters of regional brain iron distribution were found, differentiating patients and patient offspring from healthy controls and control offspring with 94% sensitivity (OR: 8.9; *p* = 0.038). Secondary analysis revealed no significant difference in brain iron distribution among BD, BPD, and ADHD diagnoses.

**Conclusion:**

Our finding of a characteristic brain iron distribution pattern in individuals diagnosed with BD, BPD, and ADHD and offspring of diagnosed individuals supports that brain iron patterns may serve as neurobiological correlates for psychiatric disorders conceptualized as spectrum conditions. Further, longitudinal studies are needed to confirm whether pattern analysis of brain iron distribution may represent a transdiagnostic biomarker for a better understanding of underlying neuropathology in psychiatric disorders.

## Graphical abstract

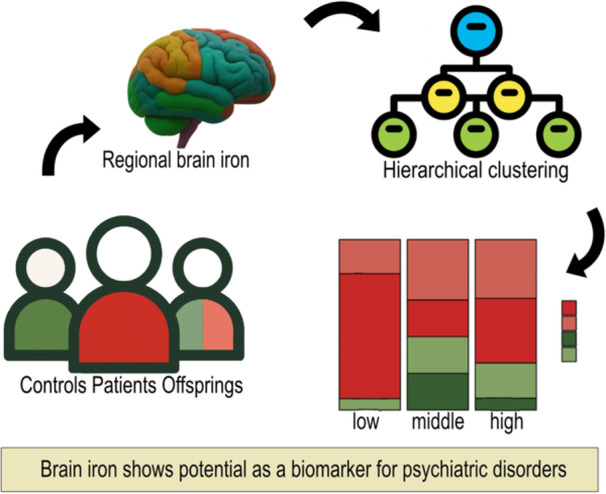

## INTRODUCTION

Common diagnostic criteria for diseases are predominantly based on categorically determining whether a specific diagnosis can be confirmed or excluded. However, recent advances in the understanding of biological traits suggest that they might exist rather on a spectrum or even continuum ranging from healthy to severely affected. This perspective has gained recognition in many fields, such as neurodegenerative diseases, or in the concept of neurodiversity.^1^, ^2^ By challenging traditional diagnostic boundaries, such a paradigm shift paves the way for identifying individuals along this spectrum before the onset of clinical symptoms. Despite these advances, the underlying pathological mechanisms driving these continuous processes remain poorly understood in many areas, and reliable biomarkers are still an unmet need.

In this context, brain non‐heme iron deposition emerges as an intriguing candidate for a non‐invasively quantifiable biomarker. Brain iron plays a critical role in the integrity and proper functioning of neural systems^3^ and was shown to affect monoaminergic neurotransmission essential for cognitive functions and emotional regulation.^4^, ^5^ Imbalanced iron metabolism has been suggested to be implicated in several psychiatric and neurodegenerative disorders^5^, ^6^, ^7^, including anxiety, depression, bipolar disorder (BD)^8^, schizophrenia,^9^, ^10^ as well as Alzheimer's and Parkinson's Disease.^11^, ^12^, ^13^ However, the vast majority of these studies focus on serum iron rather than brain iron, and peripheral iron levels do not reflect brain iron status as a consequence of blood‐brain barrier compartmentalization.^14^, ^15^

Quantification of brain iron tissue levels may be performed via non‐invasive magnetic resonance imaging (MRI) methods.^16^, ^17^ R2* mapping at MRI is an established method for quantification of brain iron, independent from vascular disease and brain volume.^18^ Brain iron has gained significance as a biomarker of brain pathologies such as neurodegeneration and neuroinflammation with relevance for clinical diagnostics.^19^, ^20^ This is supported by a concatenation of data demonstrating a central role of a well‐regulated iron metabolism for essential processes in the brain, including development and maintenance of nerve cells, neurochemical circuits, neurotransmitter synthesis, myelination, hemoglobin production, immune response, and mitochondrial function.^21^, ^22^ Iron accumulates in the brain during normal healthy aging, and this process is associated with lifestyle factors such as smoking, but also obesity.^23^

Consistently, excessive accumulation of non‐heme iron in the brain is a well‐established finding in several neurodegenerative disorders, including Alzheimer's disease (AD) and Parkinson's disease (PD).^13^, ^24^, ^25^, ^26^ Interestingly, altered brain iron may particularly reflect or interact with the inherited risk of neuropsychiatric disorder, as demonstrated for carriers of a huntingtin (HTT) trinucleotide repeat expansion or the Apolipoprotein E epsilon 4 (APOE4) allele.^27^, ^28^, ^29^ More recently, altered brain iron levels have also been reported for psychiatric and affective disorders, such as ADHD^30^, depression,^31^ and substance abuse.^32^

Pathologic processes initiated by excessively high levels of labile ferrous iron in the brain include the formation of toxic free radicals through the Fenton and Haber‐Weiss reaction, and subsequent oxidative stress, altered cell metabolism, disruptions in Ca²⁺signaling, dysregulated microglial activation, and ferroptosis.^33^, ^34^, ^35^, ^36^

These iron‐dependent molecular mechanisms have been proposed as common underlying biomechanical interactions, potentially connecting different psychiatric disorders at a mechanistic level.^37^ Although some evidence points toward an association between early brain iron deficiency and increased risk of ADHD and depression^38^, ^39^, systematic investigations into brain iron dysregulation as a shared mechanism underlying affective dysfunction, mood disorders, or psychiatric illnesses in humans remain scarce.

Traditional psychiatric diagnoses rely on descriptive approaches that define the characteristics of specific disorders without incorporating neurobiological processes into clinical assessments. The incomplete understanding of the biological mechanisms underlying psychiatric disorders highlights the need for further research to develop more precise and effective diagnostic and therapeutic strategies, where iron metabolism could represent a very promising tool.

In the current study, hierarchical clustering analysis (HCA)^40^, ^41^ was used for exploratory analysis of regional similarities in brain iron distribution, as measured by R2* magnetic resonance imaging (MRI)‐based multi‐parameter mapping (MPM).^18^ HCA does not rely on assumptions like statistical power and lacks a fixed sample size requirement,^42^ making it well‐suited for small and heterogeneous datasets, particularly when clusters may differ in size and density.^43^ Identified similarity patterns were used for characterizing divisive clusters of individuals diagnosed with common psychiatric disorders (bipolar disorder [BD], borderline personality disorder [BPD], and attention‐deficit/hyperactivity disorder [ADHD]) versus healthy controls. Although no study to date has reported a direct association between BPD and brain iron levels, the inclusion of BPD alongside BD and ADHD was motivated by the high clinical and genetic comorbidity among these conditions^44^, ^45^, ^46^ and the increasing interest in transdiagnostic approaches to psychiatry. By focusing on this constellation of frequently co‐occurring disorders, the current pilot study aims to explore whether shared or distinct patterns of brain iron distribution may point to underlying biological mechanisms. Given earlier findings supporting the role of iron in characterizing inherited risk for neuropsychiatric morbidity and suggesting that the effects of iron may be moderated by genetic risk,^27^, ^28^, ^29^ offspring of a different patient group but with the same diagnosis and controls were included in the HCA.

## METHODS AND MATERIALS

### Participants

Participants were recruited as part of a comprehensive study aimed at identifying vulnerability markers for BD, BPD, and ADHD. The study encompassed the collection of various clinical, cognitive, and affective parameters using several techniques.^47^, ^48^, ^49^, ^50^, ^51^ Patients were enlisted from outpatient programs of the Psychiatry Department of our University Hospitals and met the DSM‐IV‐TR criteria for BPD, ADHD, and BD, as a representation of frequent psychiatric disorders in adults. Diagnoses were confirmed by trained psychologists using the Diagnostic Interview for Genetic Studies^52^; additionally, the Structured Clinical Interview for DSM‐IV^53^ and the Mini‐International Neuropsychiatric Interview were conducted.^54^ For individuals diagnosed with BD, maintaining stable medication and reaching euthymic status for 4 weeks were additional requirements for inclusion. Euthymic status was defined by a Young Mania Rating Scale (YMRS) score of <6^55^ and a Montgomery‐Åsberg Depression Rating Scale (MADRS) score of <13.^56^ First‐degree relatives (offspring) from other patients diagnosed with one of these three conditions were recruited with the support of individuals attending the specialized outpatient programs at the Psychiatry Department of our University Hospitals. Prior to inclusion, all participants underwent screening for any psychiatric disorders. Healthy control participants were recruited via announcements. They were matched to the patient group regarding age, sex, handedness, and educational level. Eligibility required having no personal history of psychiatric or neurological treatment, and reporting no parental history of psychiatric disorders, as verified during the participant interview. There were no blood relatives among the recruited controls, patients, and offspring included. Ethical approval of the study was obtained and granted by the University of Geneva (CER 12‐081). All participants provided written informed consent and were compensated. In total, 92 patients, 67 patients’ offspring, 34 patients’ controls, and 39 offspring's controls were recruited. MPM data of sufficient quality were available for 39 patients, 41 patient offspring, 19 controls, and 24 control offspring.

### Procedure

Data collection was completed between 2014 and 2018 and took place at the Brain and Behavior Laboratory located at the University Medical School and the University Hospitals of Geneva. Participants had to attend three sessions. The first session included diagnostic interviews, clinical questionnaires, and blood samples. In the second session, participants completed several questionnaires to identify strategies for emotion regulation and affective disorders, as well as a working memory task. The questionnaires are detailed in the section on psychological assessments. The last session included a full structural and functional MRI assessment, including T1, diffusion tensor imaging, MPM, resting state, and a stress‐reactivity task.

### Psychological assessments

All participants completed several standardized diagnostic questionnaires on affective regulation strategies and emotional disturbances. The following four assessments were applied to quantify affective dysfunction and emotional problems in the patient, offspring, and control groups. The severity of ADHD‐related symptoms was estimated via the Adult ADHD Self‐Report (ASR), incorporating two different scores related to the core symptoms of inattention and hyperactivity/impulsivity.^57^ Anger as an emotional situational response or a dispositional quality was assessed as state anger, trait anger, and anger expression using The State‐Trait Anger Expression Inventory (STAXI).^58^ Individual strategies for cognitive emotion regulation, both adaptive and maladaptive, were identified by the Cognitive Emotion Regulation Questionnaire (CERQ).^59^ Encoding styles related to schizotypal traits and impulsivity were assessed using the 24‐item Encoding Style Questionnaire (ESQ), which measures the tendency to interpret environmental cues either hastily through pre‐existing schemas or more conservatively.^60^ Working memory was assessed using a reversal digit span test.

### Iron imaging

The multiparametric mapping (MPM) sequence includes a set of quantitative measures to highlight intrinsic characteristics of brain tissue indicative of myelin, iron, and tissue water content. R2* maps used in this study correlate with the iron content. Participants underwent a 3 T head MRI scan (Trio, Siemens Healthineers, Erlangen, Germany) with a 32‐channel head coil. A sagittal 3D isotropic T1 Magnetization‐Prepared Rapid Acquisition Gradient Echo (MPRAGE) sequence was performed using the following parameters: repetition time (TR) = 1900 ms, echo time (TE) = 2.27 ms, voxel size = 1 x 1 x 1 mm3, flip angle (fa) = 9o, 1 average. In addition, a multi‐echo FLASH sequence was carried out for MPM covering the entire brain using the following parameters: TR = 24 ms; TEs = 2.16/4.75/7.34/9.93/12.52/15.11/17.7/20.29 ms; voxel size = 1 x 1 x 1 mm3, (fa) = 6o, 1 average. The brain multimodal data were analyzed with the hMRI toolbox^17^ running on standard statistical software, SPM12^61^, using MATLAB (version R2022a, Mathworks, Inc., US), as described in detail elsewhere.^17^ Subject motion was addressed in the hMRI toolbox through a multi‐faceted correction strategy, including inter‐scan correction using sensitivity mapping, RF inhomogeneity correction, and retrospective motion correction during reconstruction.^17^ R2*maps (s‐1) were calculated using an ESTATICS model with ordinary least squares fit. The ESTATICS model was introduced by Weiskopf et al.^62^ to efficiently estimate the effective transverse relaxation rate R2* from an MPM acquisition protocol. T1‐weighted images were segmented using FreeSurfer via their Aseg probabilistic atlas. Then, T1‐weighted anatomical images were registered to the R2* maps using the MINC toolkit. Statistics were reported for 21 cortical region‐of‐interest (ROI) (encompassing 29.32% of the whole brain standard space), as derived from gyral‐based cortical parcellation of bihemispheric ROIs as defined by the FreeSurfer's Desikan–Killiany (DK) atlas.^63^

### Statistical analysis

Statistical analyses were performed using R Statistical Software (v4.1.2).^64^ For data visualization, the R packages ggplot2 (v3.3.5)^65^, ggstatsplot (v0.9.1)^66^, and pheatmap (v1.0.12)^67^ were used. As input for the hierarchical clustering algorithm, we used the 21 ROIs of the DK‐atlas, which exhibited sufficient measurement quality of iron levels.

#### Data imputation

Before hierarchical clustering, the dataset's missing values were imputed using the Random Forest algorithm, which is implemented in the R package missForest (v1.5).^68^ This algorithm is based on decision trees and bootstrap aggregation to predict and fill in missing values. It demonstrated good performance except for outcome‐dependent missingness or significant skewness, both of which were not present in our data.^69^

#### Clustering and quality of clustering assessment

We ran a divisive analysis (DIANA)^70^ based on hierarchical clustering to define brain systems with potential variation in terms of iron deposition. DIANA works in a top‐down manner, starting with a single cluster that includes all observations. To ensure consistency in the clustering results, an additional agglomerative nesting clustering analysis was performed (AGNES).^70^ Unlike the DIANA algorithm, AGNES considers each observation as a single‐element cluster and combines single clusters into bigger clusters. We ran both analyses in an agnostic fashion. Namely, the input information included only the regional brain MPM values representing the regional iron levels measured in parts per million (ppm). The resulting clusters from both methods were compared through the Manhattan distance.^70^ Compared to further dissimilarity metrics (e.g., Euclidean distance), the Manhattan index results are less sensitive to outliers and show superior performance in brain imaging clustering.^71^, ^72^ Our clustering procedures followed the pipeline proposed by Kaufman and Rousseeuw^70^, implemented and extended in the R‐package Cluster (v2.1.2).^73^ The goodness of the clustering measure was calculated using the clusGap() function of the Cluster Package.^73^ This function selects the optimal number of clusters by maximizing the gap statistics and comparing the total intra‐cluster variation for different numbers of clusters with their expected values under the null reference distribution of the data.

The clustering quality was assessed using the silhouette coefficient overall and separately for each cluster, as well as using the Calinski‐Harabasz index and the Davies‐Bouldin index.^74^, ^75^ We additionally applied a Principal Component Analysis to visualize the clustering solution in reduced‐dimensional space and to qualitatively assess the spatial relationship between clusters.^43^

#### Differences in brain iron levels across regions and clusters

Post‐hoc contrast variances between clusters and brain regions were assessed with the Conover‐Iman Test included in the R‐package Conover.test (v1.1.5).^76^ This non‐parametric test is suitable for comparing multiple groups with unbalanced sample sizes. The obtained p‐values were adjusted for multiple group comparisons using the Benjamini–Hochberg correction. To control for Type I error, the significance level was adjusted for a two‐tailed test (α/2).^76^

#### Demographics and hypothesis testing

In the next step, we added demographic information (age, sex, body mass index [BMI]) and group assignment (patient, patient offspring, control, control offspring) for each participant. Diagnostic information was additionally included for the patient group. The participants were identifiable by their subject numbers. Clusters were tested for significant differences in age, sex, and BMI. Statistical tests were selected based on the fulfillment of their underlying assumptions (either *t*‐test, Chi‐squared test, Kruskal‐Wallis test, or ANOVA). For the Kruskal‐Wallis test, the coin package (v1.4.2)^77^ was used. To test our first hypothesis, which assumed that clustering of regional iron levels could distinguish patients and individuals at risk from controls and control offspring, we created a binary factor with the levels “patients/individuals at risk” and “controls/control offspring.” The absolute number of patients/offspring and controls/control offspring within each cluster was then compared for significant differences using Fisher's exact test, which is appropriate for small and unequal sample sizes^78^, and validated with a chi‐square test. In this way, we identified clusters that contained significantly more patients and individuals at risk. Next, we calculated the odds ratio that a patient is classified as either “at risk” (meaning belonging to a cluster with more patients and patient offspring) or “control” based on the clustering of regional iron levels. The odds ratio assesses the strength of association between these two groups and the identified clusters, or in other words, the relationship between exposure (specific iron patterns) and outcome.

To test our second hypothesis, assuming that patient clusters would be associated with established psychiatric diagnoses, we ran a similar analysis and calculated the odds ratio per cluster for each given diagnosis (BPD, ADHD, and BD). This analysis enabled us to determine whether the algorithm could identify different psychiatric diagnoses based on regional iron distributions.

To test our third hypothesis, assuming that psychometric traits, such as emotion and self‐regulation, differ among the clusters, we employed one‐way ANOVAs or Kruskal–Wallis tests. The selection of statistical tests was based on whether the data met the assumption of normality. To test our third hypothesis, which suggested that psychometric traits like emotional regulation and self‐regulation differ between clusters, we employed one‐way ANOVAs or Kruskal–Wallis tests, depending on the data distribution. The quantitative score of the psychometric inventory served as the dependent variable, while group was treated as the independent variable.

## RESULTS

### Missing data and data imputation

The extraction of MPM values for all regions of each subject was sometimes hindered by the total data quality resulting from movements and other factors. We limited our focus to brain regions containing more than 100 observations over all subjects to ensure a sufficient sample size for data imputation. By this criterion, we selected the following 20 brain regions: Caudate, Putamen, Thalamus, Cuneus, insular cortex, medial and lateral orbitofrontal cortex, superior frontal and rostral middle frontal cortex, rostral anterior cingulate cortex, superior, middle, and inferior temporal gyrus, fusiform gyrus, precentral gyrus, precuneus, superior and inferior parietal lobe, pericalcarine cortex, lateral occipital cortex, and lingual gyrus. In terms of the entire DK atlas, these regions account for 29.32% of the total brain volume. For those regions, missing values were imputed by applying the random forest algorithm. The pattern of missing data is shown in the Figure S1.

### Divisive hierarchical clustering of brain regional MPM values

**Figure 1:**
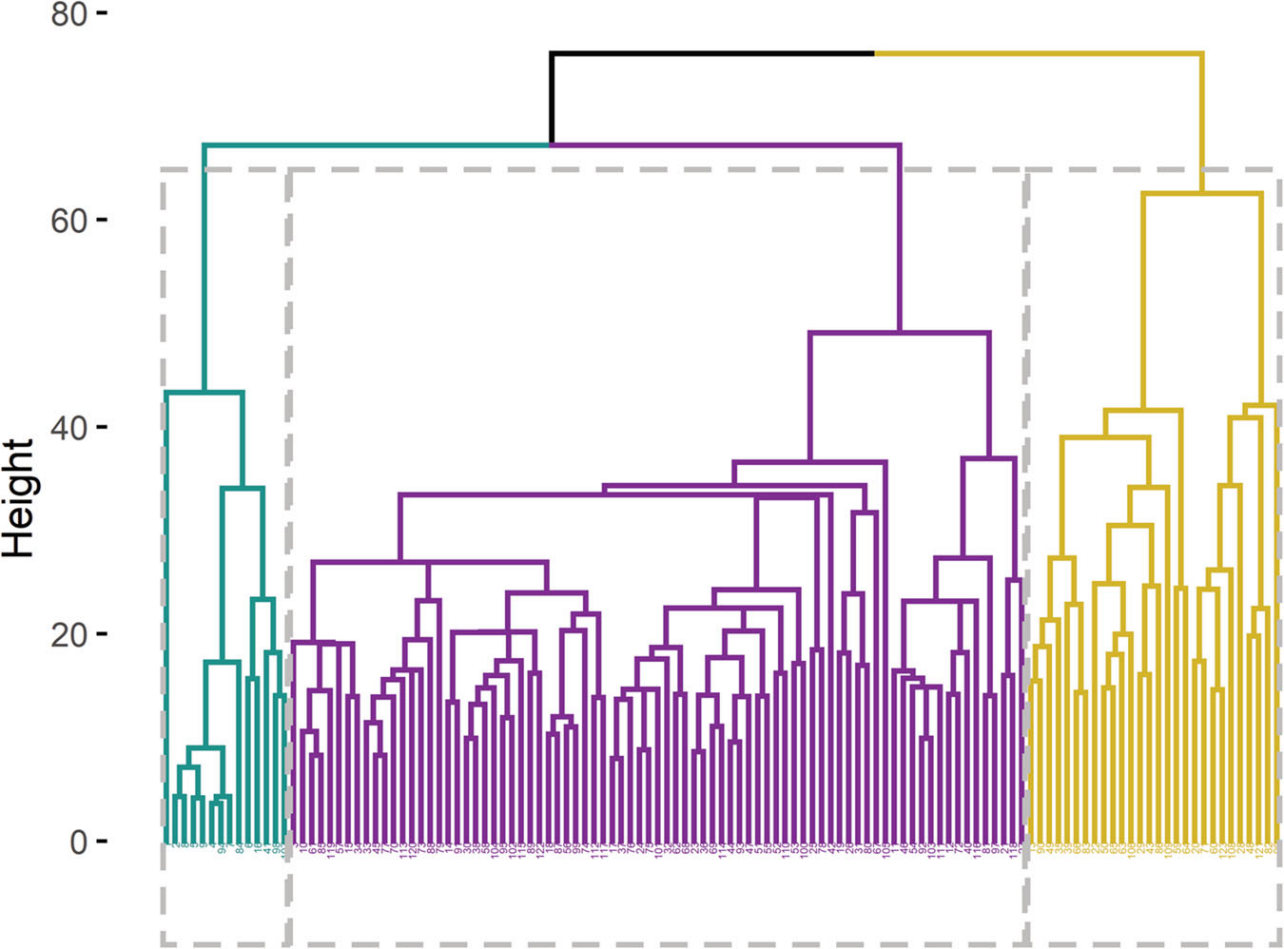
Hierarchical clustering dendrogram. Clusters were derived using the DIANA algorithm, with data assigned into three clusters based on MPM values from distinct brain regions. The *X*‐axis represents subjects. Cluster 1 = purple, *N* = 15; Cluster 2 = green, *N* = 82; Cluster 3 = yellow, *N* = 26.

**Figure 2:**
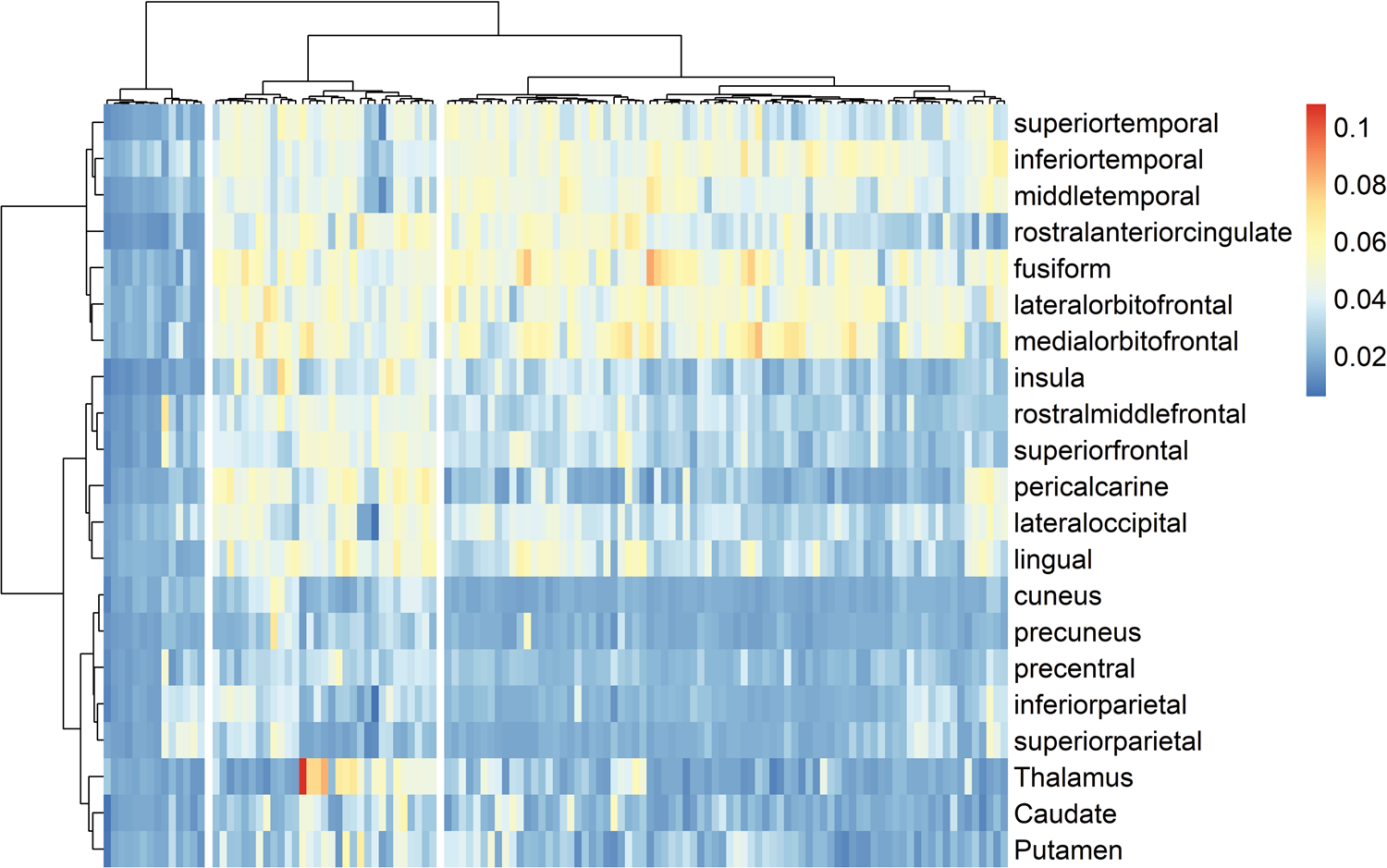
Cluster‐specific iron levels across brain regions. Clusters were derived using the DIANA hierarchical clustering algorithm, with data assigned into three clusters based on R2* values from distinct brain regions. Blue indicates lower R2* values, yellow and red indicate higher R2* values.

The optimal number of clusters, as indicated by the gap statistics, was three. After running DIANA, we obtained three clusters with 15, 79, and 29 subjects, respectively. The application of AGNES as a clustering method exhibited strong consensus. However, a minor dissimilarity arose in the allocation of one single subject, with AGNES assigning it to Cluster 2 while DIANA assigned it to Cluster 3. The clustering results of DIANA are visualized in Figures 1 and 2. Overall, the mean R2* values (as indicators of iron) between clusters were significantly different (*F*(2, 120) = 124, *p* < 0.001). Tukey's HSD Test for multiple comparisons found lower MPM values in Cluster 1 than in Cluster 2 (*p* < 0.001, 95% CI = [0.010, 0.015]) and Cluster 3 (*p* < 0.001, 95% CI = [0.016, 0.021]); and further, Cluster 2 had lower R2* values than Cluster 3 (*p* < 0.001, 95% CI = [0.004, 0.008]).

### Evaluation of clustering results

With an average silhouette coefficient of 0.290, the overall clustering solution indicated only a modest degree of cluster separation. However, examining the silhouette coefficient for each cluster revealed relatively good cohesion and separation for Cluster 1 (0.418), while Cluster 2 (0.290) and particularly Cluster 3 (0.107) demonstrated weaker internal structure and greater overlap with each other. The Calinski–Harabasz index reached a value of 35.60, suggesting a reasonable degree of between‐cluster separation relative to within‐cluster dispersion. The Davies–Bouldin index was 1.41, which indicates some overlap among clusters, without specifying which clusters. To further assess the cluster structure, a principal component analysis (PCA) was conducted, confirming that Cluster 1 was well separated, while Clusters 2 and 3 showed substantial overlap (Figure S2).

### Differences in brain regions across clusters

**Figure 3:**
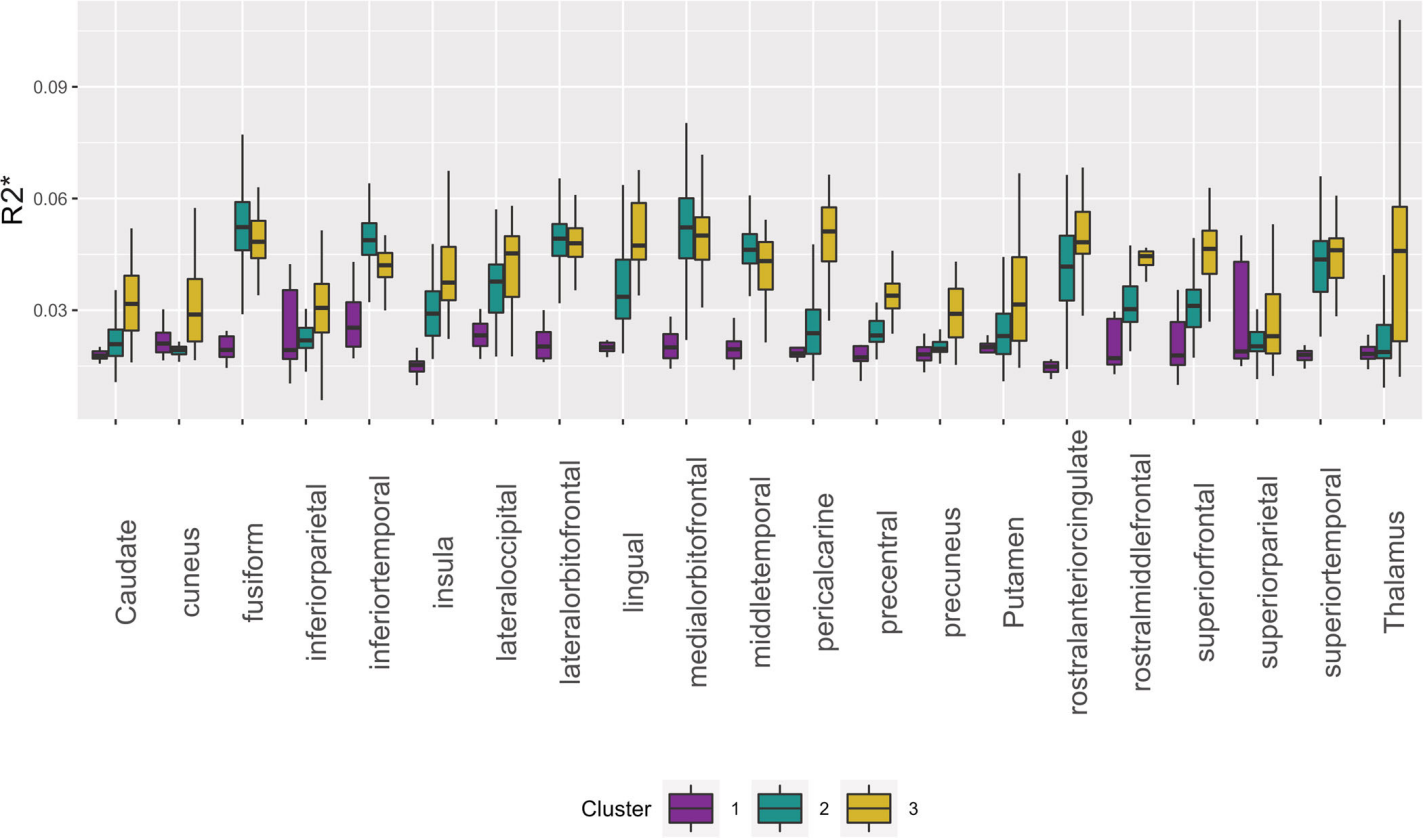
Iron level differences between clusters. Boxplots illustrating the R2* derived brain iron (ppm) for individual brain regions. Cluster 1 = purple. Cluster 2 = green, Cluster 3 = yellow.

The Conover–Iman tests yielded significant differences between clusters for almost all comparisons and brain regions. Regions that did not reach statistical significance when comparing Cluster 1 and Cluster 2 were the inferior parietal gyrus, precuneus, putamen, and thalamus. Further, no statistical significance was observed between Clusters 2 and 3 for the fusiform gyrus, lateral orbitofrontal gyrus, medial orbitofrontal gyrus, and superior temporal gyrus. No significant differences were found for the superior parietal gyrus across all clusters. The results are depicted in Figure 3 and detailed in Table 1. As a result, our examination of MPM values across 21 brain regions identified three distinct clusters, each showing significant disparities in brain iron spatial distribution. The 21 bilateral brain ROIs, as depicted in Table 1, represent 29.13% of the Desikan–Killiany atlas in terms of volume.

**Table 1:** MPM value differences between DIANA clusters.

*Region*	*Δ between 2 and 1*		*Δ between 3 and 1*		*Δ between 3 and 2*	
	Rank *r*	*p* value	Rank *r*	*p* value	Rank *r*	*p* value
Caudate	2.24	0.013*	5.07	<0.001*	4.51	<0.001*
Cuneus	−2.09	0.019*	2.16	0.024*	5.84	<0.001*
Fusiform gyrus	5.66	<0.001*	3.77	<0.001*	−1.76	0.045
Inferior parietal gyrus	0.13	0.449	2.61	0.008*	3.64	<0.001*
Inferior temporal gyrus	6.30	<0.001*	2.17	0.016*	–4.90	<0.001*
Insular cortex	4.80	<0.001*	7.01	<0.001*	4.07	<0.001*
Lateral occipital gyrus	3.09	<0.001*	5.31	<0.001*	3.77	<0.001*
Lateral orbitofrontal gyrus	7.02	<0.001*	5.67	<0.001*	−0.73	0.231
Lingual gyrus	4.74	<0.001*	7.43	<0.001*	4.75	<0.001*
Medial orbitofrontal gyrus	6.94	<0.001*	5.05	<0.001*	1.54	0.063
Middle temporal gyrus	5.30	<0.001*	2.90	0.004*	−2.56	0.006*
Pericalcarine cortex	1.78	<0.039*	6.54	<0.001*	7.25	<0.001*
Precuneus	1.33	0.093	5.34	<0.001*	6.08	<0.001*
Precentral gyrus	3.18	<0.001*	7.50	<0.001*	6.85	<0.001*
Putamen	0.90	0.184	3.39	<0.001*	3.79	<0.001*
Rostral anterior cingulate gyrus	4.96	<0.001*	6.13	<0.001*	2.58	0.006*
Rostral middle frontal gyrus	3.38	<0.001*	7.51	<0.001*	6.60	<0.001*
Superior frontal gyrus	2.82	0.003*	7.13	<0.001*	6.76	<0.001*
Superior parietal gyrus	0.14	0.445	0.52	0.453	0.58	0.842
Superior temporal gyrus	5.45	<0.001*	5.21	<0.001*	−0.61	0.272
Thalamus	1.64	0.052	4.22	<0.001*	4.06	<0.001*

*Note*: Statistical comparisons derived by the Conover‐Iman test between Cluster 2–Cluster 1; Cluster 3–Cluster 1; Cluster 3–Cluster 2. All results for the 21 brain regions included were adjusted for multiple comparisons by Benjamin‐Hochberg correction. H0 was rejected if *p* < =alpha/2. Significant differences are indicated by*.

### Demographics and risk assessment (odds ratio)

**Figure 4:**
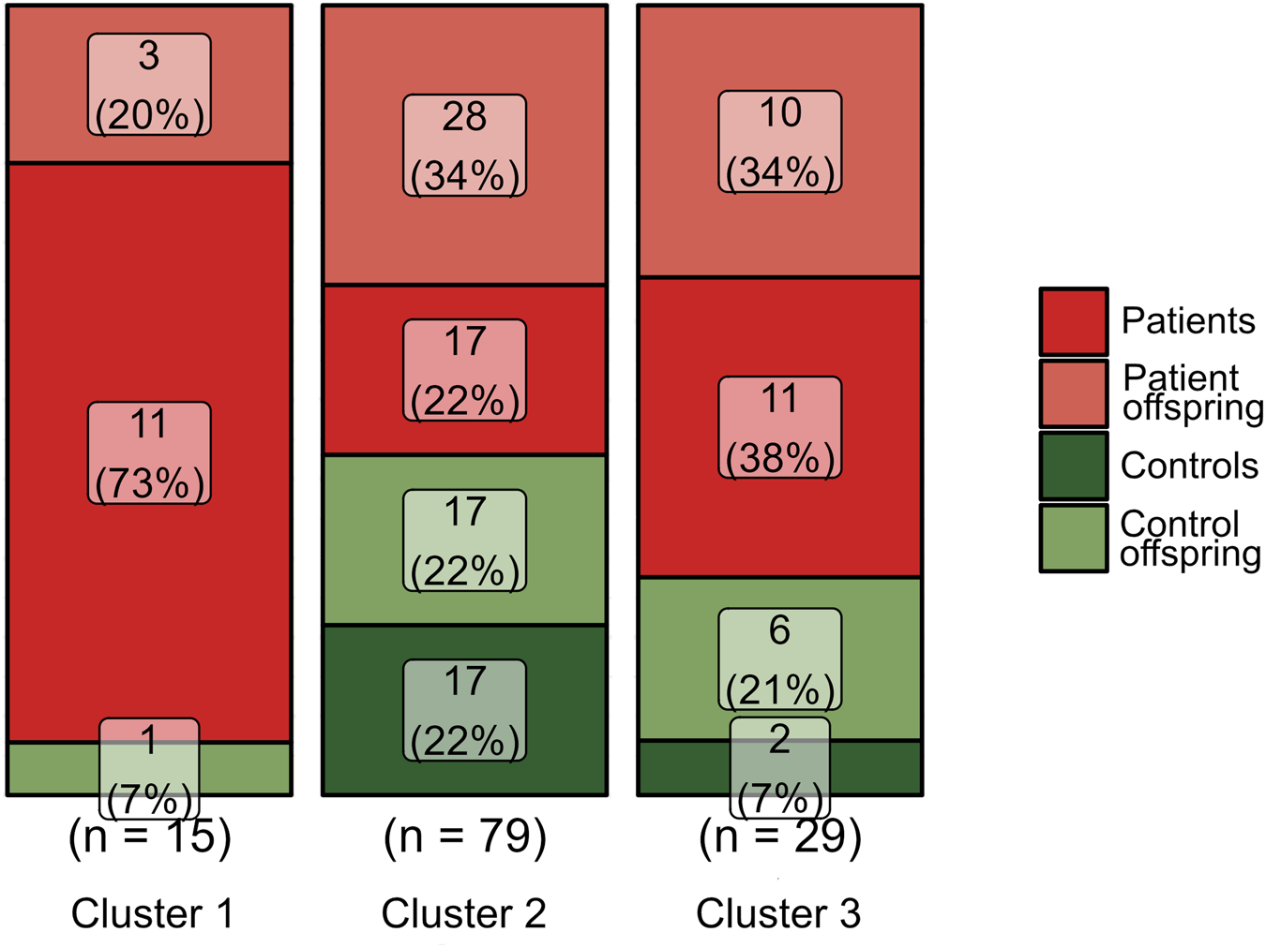
Cluster‐wise group composition. Absolute number and cumulative percentage of patients/offspring and controls/control offspring within each cluster. To calculate differences between clusters, patients and patient offspring were grouped as one level of a binary factor, while controls and control offspring constituted the other level.

DIANA clusters did not show significant age differences (Kruskal‐Wallis Test: *χ²* = 0.053, *df* = 2, *p* = 0.975) or differences in sex distribution (Chi‐squared Test: *χ²* = 2.919, *df* = 2, *p* = 0.233) and no differences for BMI (ANOVA: *F* = 0.865, *p* = 0.424). More details about demographic and clinical characteristics by the DIANA cluster are provided in Table S1. Next, the subjects were assigned to a binary factor: 80 patients (mean age = 27 years, 64% females) and patient offspring (mean age = 20 years, 59% females) were classified as level 1, while 43 control subjects (mean age = 31 years, 63% females) and patient offspring (mean age = 20 years, 50% females) were classified as level 2. Fisher's exact test, which suits small and unequal sample sizes, revealed significant differences between the three clusters in the proportional distribution of patients/offspring and controls overall (*p* = 0.015). Applying a Chi‐square‐test provided comparable results (*χ²* = 8.12, *df* = 2, *p* = 0.02). Next, the percentage proportion of patients/offspring vs. controls/offspring was compared within every single cluster (Figure 4). For Cluster 1 and Cluster 3, the percentage proportions were statistically different (Cluster 1: *χ²* = 11.27, *df* = 1, *p* < 0.001; Cluster 3: *χ²* = 5.54, *df* = 1, *p* = 0.019), while both clusters contained more patients/offspring than controls. This was not the case for Cluster 2 (*χ²* = 1.76, *df* = 1, *p* = 0.185). These findings demonstrate a different proportionality between patients and controls in two of the three clusters. Accordingly, the hierarchical clustering algorithm successfully identified iron patterns more strongly associated with individuals at risk or patients.

**Figure 5:**
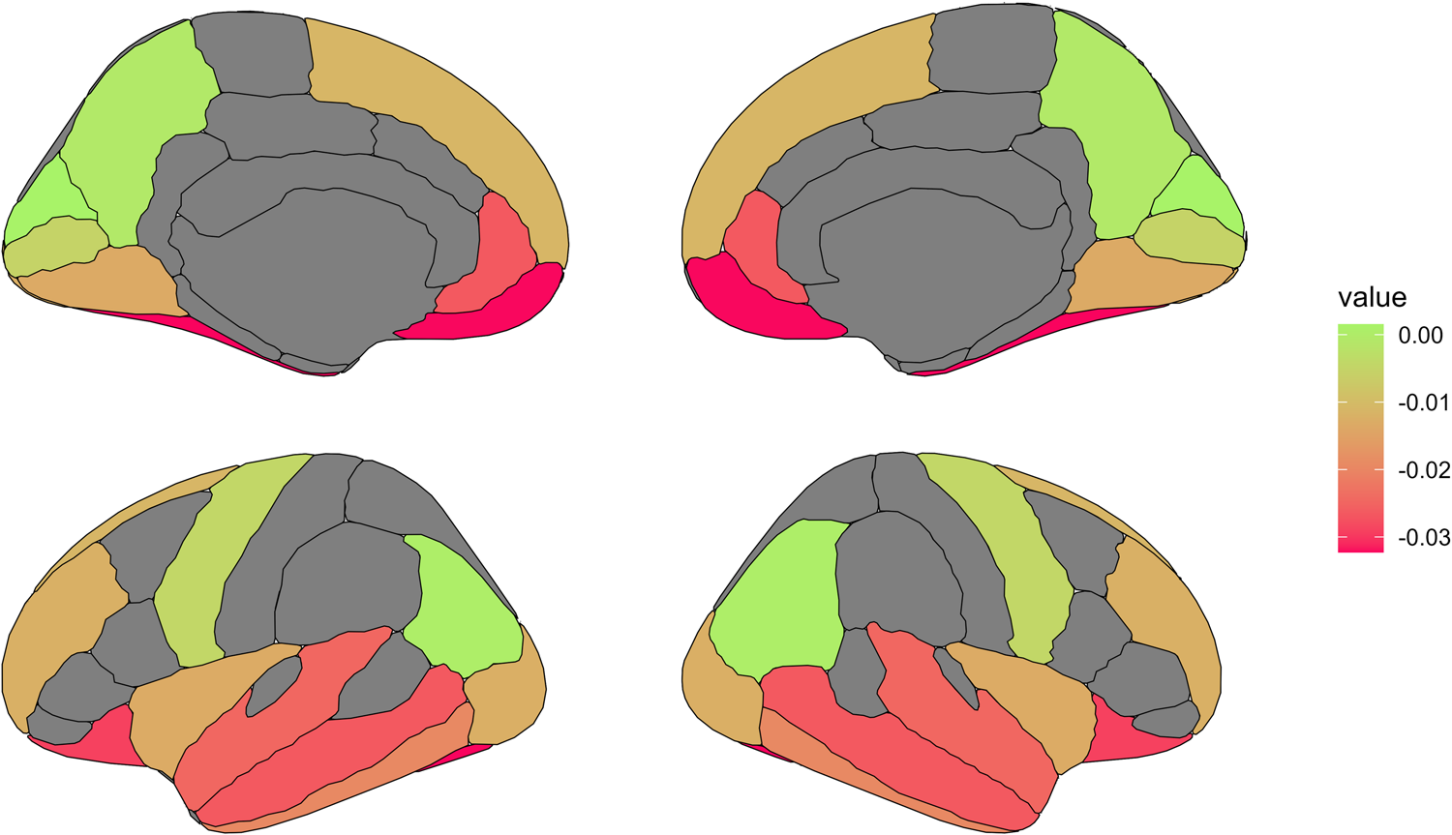
Iron differences between clusters. Median differences of brain iron (ppm) per region between Cluster 2 and Cluster 1 are shown.

To evaluate the strength of the association between exhibiting a specific distributional pattern of iron in the brain and the likelihood of belonging to the group of individuals at risk, we calculated the odds ratio for these outcomes (specifically, Cluster 1 or Cluster 3). The odds ratio for Cluster 1 was 8.9 (95%‐CI: 1.1–70.2, *p* = 0.038), indicating a strong association between exhibiting a Cluster 1 regional iron distribution and being a patient. However, subjects with Cluster 3 iron distribution had an odds ratio of 1.5 (95% CI: 0.6–3.8, *p* = 0.340), suggesting a weak but non‐significant association. Cluster 1 exhibited a sensitivity of 94%, indicating a strong capability of correctly identifying persons with the respective iron pattern as positive cases or at‐risk individuals. This high sensitivity ensures that individuals with the specific iron pattern are reliably identified as persons with increased risk for psychiatric disease. However, it was associated with a rather low specificity of 39%, which suggests that some negative cases are misclassified as persons at risk. Regions in Cluster 1 with significantly lower iron levels compared to Cluster 2 are shown in Figure 5.

### Diagnoses in the patient group

**Figure 6:**
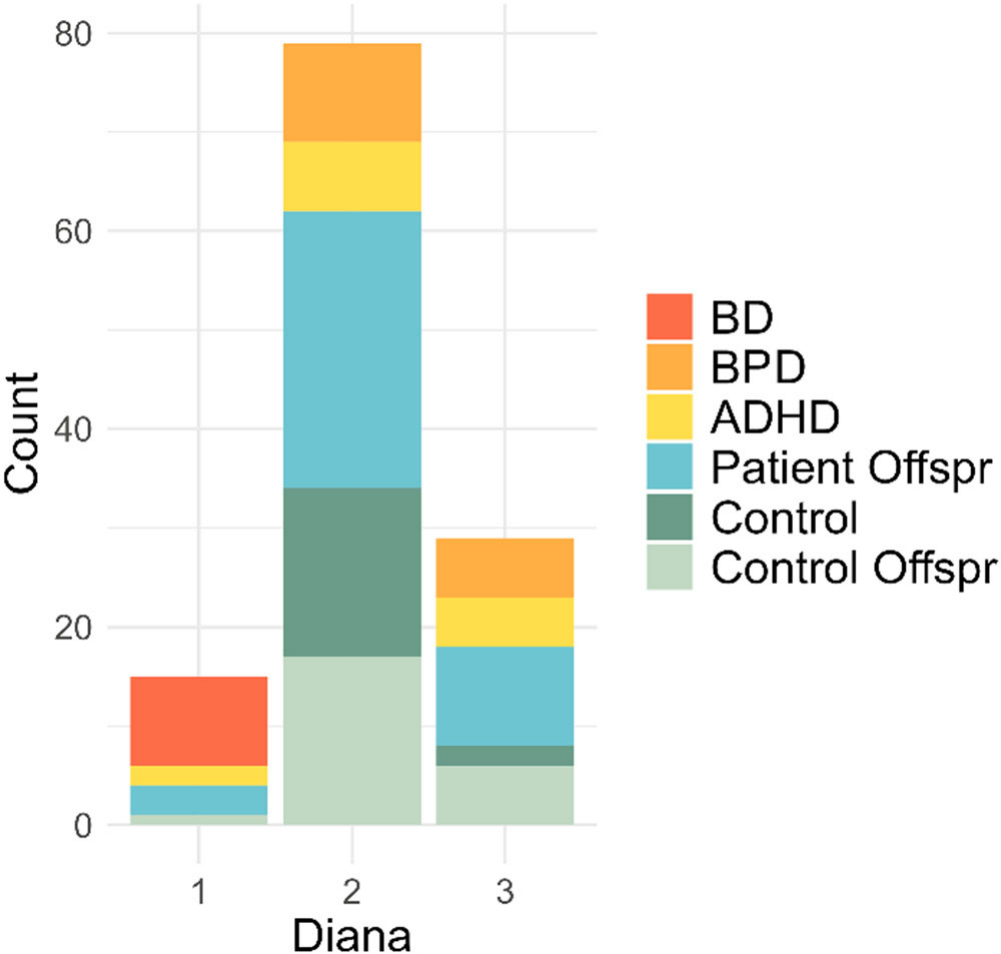
Frequency of pathologies within clusters. ADHD, attention deficit/hyperactivity disorder; BD, bipolar disorder; BPD, borderline personality disorder; Offspr, offspring.

Our second hypothesis assumed relationships between specific psychiatric diagnoses and their prevalence in certain clusters. To test whether one of the diagnoses within the patient group occurs more frequently in one of the clusters, we employed Fisher's exact test, which yielded nonsignificant results (*p* = 0.754, odds ratio = 0.67). Accordingly, in statistical terms, the frequency of diagnoses was similar across the clusters, and regional iron quantification did not yield Clusters that represent distinct traditional psychiatric diagnoses. Interestingly, however, only BD patients were found in Cluster 1, yet none of the patients with a diagnosis of BPD (see Figure 6 and Table S1). The relatively small cluster size may have hindered the achievement of statistical significance.

### Psychometric traits

Our third hypothesis proposed differences among the clusters in the distribution of psychometric traits, such as emotion regulation and anger management. However, no differences between clusters were found for depressive symptoms (MADRS, *H*(2) = 2.93, *p* = 0.231); hyperthymic symptoms (YMRS, *H*(2) = 0.06, *p* = 0.969); anger as a trait (STAXI, *H*(2) = 4.84, *p* = 0.089); encoding style questionnaire (ESQ, *F*(2, 115) = 1.30, *p* = 0.275); inattention (ASRS, *H*(2) = 0.82, *p* = 0.662); hyperactivity/impulsivity (ASRS, *H*(2) = 1.03, *p* = 0.598); and working memory (*H*(2) = 0.30, *p* = 0.860).

### Medication, substance use, and alcohol consumption in relation to brain iron

Cluster 1 exhibited the highest rates of both substance use and alcohol consumption (see Tables 3–4). However, this same cluster displayed the lowest brain iron values, despite evidence that substance use, especially alcohol and stimulants, is typically associated with increased brain iron accumulation. Previously, alcohol use disorder has been linked to elevated brain iron in the dorsal striatum.^79^, ^80^ Substance use disorders like cocaine addiction have also been associated with iron accumulation in the globus pallidus and other basal ganglia structures ^81^, ^82^. However, our cohort is relatively young (range 15–58 years, mean age: 24 years, median age: 22 years; see Table S1 for further details), whereas most studies reporting alcohol‐ or substance‐related iron accumulation involve middle‐aged or older populations. With respect to medication exposure (Table S2), no agents known to promote iron accumulation in the brain were included. Current evidence does not indicate that these medications alter cerebral iron levels.

## DISCUSSION

We employed divisive hierarchical clustering to analyze brain iron levels across 21 brain regions in patients, offspring of unrelated individuals with the same diagnosis (patient offspring) as mental disease at‐risk persons, as well as matched control participants and control offspring. Our goal was to investigate whether patterns of cerebral iron distribution could effectively differentiate patients with transdiagnostic mental disorders and at‐risk individuals from healthy controls into distinct clusters. Three distinct clusters were identified by the hierarchical clustering algorithm solely based on iron levels, independent of any diagnostic or demographic information. The clusters varied considerably in size, with the smallest containing 15 subjects (Cluster 1), the medium‐sized cluster containing 28 subjects (Cluster 3), and the largest cluster containing 80 subjects (Cluster 2). No differences in sex or age were found between clusters. Subjects in Cluster 1 exhibited lower non‐heme iron levels across nearly all investigated regions compared to the other clusters, while those in Cluster 3 showed higher levels in several brain regions. The ratio of patients/offspring as compared to controls was higher in both Cluster 1 and Cluster 3. Accordingly, two types of cerebral iron distribution patterns were identified, which were associated with a higher likelihood of belonging to the patient and at‐risk groups. These findings indicate that individuals with either low or high non‐heme iron levels were more likely to be associated with the patient or patient offspring groups. More specifically, this finding was based on MPM values measured across multiple regions encompassing 29.32% of the brain. The strength of the association was evaluated using odds ratios. For Cluster 1, the odds ratio was significantly elevated at 8.9, indicating a strong association between a Cluster 1‐like brain iron pattern and an increased risk of having a psychiatric condition. This was reflected in a very high sensitivity of 94%, meaning the model effectively identifies psychiatric patients and offspring at risk. However, it was accompanied by a relatively low specificity, suggesting that many healthy individuals could be incorrectly classified as having the condition. For Cluster 3, the odds ratio was lower and non‐significant. In summary, our first hypothesis was confirmed: we identified two distinct clusters with higher proportions of patients and at‐risk individuals compared to controls, and one of these clusters demonstrated high sensitivity. This suggests that brain iron distribution patterns may function as indicators of increased vulnerability to impaired mental health. Regarding our second assumption, the assignment of subjects to clusters using a hierarchical clustering algorithm did not allow differentiation between the given psychiatric diagnoses. In other words, individuals with different diagnoses were present in the clusters obtained by DIANA. Notably, almost all individuals diagnosed with BD were exclusively found within Cluster 1. In contrast, no patients with a diagnosis of BPD were found in this cluster (Figure 6). This observation did not reach statistical significance, possibly due to the small sample size, and may therefore warrant further investigation in future studies. Regarding our third assumption, no differences in psychometric traits were observed between clusters. Consequently, variations in iron distribution patterns were not linked to specific emotional difficulties or behavioral abnormalities.

Psychiatric diseases, including those from affective, psychotic, and personality disorder categories, may exist as continuous phenomena rather than binary traits.^83^ While in our study, low and high iron did not distinguish between traditional diagnostic categories, we found a group with a pattern of reduced iron that was associated with an increased risk of psychiatric disorders, particularly for BD. In addition, a distribution pattern of higher cortical iron also showed a tendency for increased risk; however, internal cohesion within this cluster was weak. Nevertheless, our results indicate that iron may represent a pathological process involved in the neurobiology of various mental health conditions and disorders. Moreover, in this study, the iron distribution pattern appeared to follow a specific distribution encompassing regions relevant to emotion processing, such as ventromedial PFC and the temporal lobe.^84^ While we have shown that cognitive emotion regulation processes, requiring dorsolateral prefrontal activity, might differ between these clinical presentations,^48^ we have also shown in this population a transdiagnostic marker of emotion dysregulation with increased BOLD signal variability in similar regions.^50^ Our findings here reinforce the hypothesis of a common impairment in initial stages of emotion detection for some patients across disorders and especially for BD, although no clinical correlation was found in the battery of questionnaires used in this study.

In line with our findings, previous studies have yielded results highlighting cerebral iron as a significant biomarker for psychiatric disorders and emphasizing its relevance for further research aimed at a better understanding of underlying neuropathology in brain disorders. For example, low subcortical iron levels were predictive of cognitive‐affective dysfunction in depressed patients^85^ and more prevalent in psychotic spectrum disorders^86^. As molecular underpinnings between brain iron deficiency and psychiatric symptoms, impaired monoaminergic neurotransmission, NMDA‐receptor dysfunction, and thyroid hormone dysfunction have been proposed.^39^

On the other hand, subjects in Cluster 3 had considerably higher brain iron levels across several brain regions. To date, high brain iron levels have been associated with neurodegenerative rather than psychiatric disorders, beyond a few studies reporting associations between brain iron accumulation and depression, anxiety disorder, or schizophrenia. Previously, non‐heme brain iron correlated positively with the severity of depression^87^, and abnormally elevated brain iron levels as found in late‐life depression patients were even correlated with the duration and progress of the disease.^31^ Another study demonstrated elevated iron levels, decreased ferritin levels, and an increased iron‐to‐ferritin ratio in the prefrontal cortex of schizophrenic patients. The authors suggested a pathophysiological connection between disrupted cortical iron biology and schizophrenia, supporting the use of in vivo iron imaging as a tool for monitoring clinical progression and treatment responses.^88^ Other studies in schizophrenia patients revealed elevated iron levels in the putamen and thalamus.^89^, ^90^ Further, brain iron accumulation was shown to disrupt functional networks^91^ and white matter microstructure of structural networks^92^ essential for cognitive and affective functions in older individuals. This aligns with the observation that myelin content inversely correlates with iron levels in aged individuals.^93^ Crucially, iron was found to interact with pathological proteins, such as APOE4, which are commonly linked to neurodegenerative diseases.^27^ Moreover, progression of Alzheimer's Disease was driven by the spatial colocalization of brain iron deposits with Aβ‐plaques.^94^ Consistently, myelin abnormalities have been found as well in psychiatric patients^95^, ^96^, raising the question of whether correcting iron imbalances in conjunction with conventional therapies can enhance treatment outcomes.

Current psychiatric classifications relying upon descriptive approaches are facing difficulties and ambiguities when diagnosing psychiatric diseases. There is considerable overlap and shared biological features among distinct psychiatric disorders, indicating a lack of clear natural boundaries in defining their presence or absence, as many exist on a continuum.^97^ The present work highlights brain iron levels as a potential diagnostic marker to identify and characterize subtypes across psychiatric diagnostic categories, similar to previous attempts in neurodegenerative disease.^20^

### Limitations and future directions

There are several limitations in this study. Firstly, due to poor data quality for some subjects, not all R2* values across all brain regions from all subjects could be used in the algorithm. Due to the low quality of the data, we considered only about 30% of the total brain volume for the analysis. Nevertheless, we were able to include several cortical regions relevant to the investigated psychiatric disorders. Secondly, the levels of iron reported here might change as a function of the brain parcellation. However, in defense of Desikan‐Killiany's atlas, the chosen parcellation is anatomically grounded. Thirdly, recent evidence suggests that MRI signals may not always align with absolute iron concentrations, especially when ferritin levels are low but iron is elevated, as this alters the magnetic properties contributing to the MRI signal.^88^ Fourthly, hierarchical clustering may be sensitive to noise and outliers, potentially leading to suboptimal clustering results. Therefore, it will be important to replicate these findings in an independent sample. Also, the clusters have been considerably different in size, which limits statistical comparisons between clusters. Finally, regarding the participants, we did not statistically control for other factors potentially impacting iron levels, such as medication, alcohol consumption, smoking, and being overweight.

Further investigations should continue to explore the potential of the relationship of brain iron levels in bridging the gap between brain and mental health, for example, by integrating brain iron measurements with additional biological and clinical data in longitudinal studies for a better understanding of individual transdiagnostic risk and resilience factors.

## CONCLUSION

The finding that categorization based on brain iron levels provides additional insights beyond psychometric traits underscores its potential as a biomarker for assessing the risk of psychiatric disorders and for prospective applications in early mental health prevention. Further research is needed to explore the significance of brain iron levels in improving diagnostic accuracy and developing personalized treatment options.

## AUTHOR CONTRIBUTIONS

**Rebecca Christina Coray:** methodology, data curation and analysis, writing – original draft, visualization. **Jatta Berberat:** formal analysis, data curation and analysis, software, writing – review and editing. **Sonja Maria Kagerer:** conceptualization, review of the statistic strategy, writing – review and editing. **Lopez Julian Gaviria:** formal analysis, data management and curation. **Nader Perroud:** study implementation, data collection. **Camille Piguet:** conceptualization, funding acquisition, study implementation, data collection, supervision. **Paul Gerson Unschuld:** development of data analysis and MRI quantification strategy, funding acquisition, supervision, writing. All authors critically revised the manuscript.

## CONFLICT OF INTEREST STATEMENT

The authors declare no conflicts of interest.

## ETHICS APPROVAL STATEMENT

The protocol was accepted by the ethical committee on human research in Geneva (CER 13‐081).

## PATIENT CONSENT STATEMENT

All participants provided written informed consent.

## CLINICAL TRIAL REGISTRATION

N/A.

## Supporting information

Supplementary Figure 1: Missing data pattern. The missingness appears to follow a systematic structure along the diagonal, suggesting that the data is not Missing Completely At Random (MCAR). Since the missingness is dependent on observed data (e.g., specific brain regions with higher noise levels in imaging), missing data is assumed to be Missing At Random (MAR). Supplementary Figure 2. PCA projection with three clusters*. Data points are colored by cluster assignment (Cluster 1 = red, Cluster 2 = green, Cluster 3 = blue). Ellipses represent the 95% confidence interval for each cluster in the two‐dimensional principal component space*. Supplementary Table 1: Demographic Table. Supplementary Table 2: Medication per DIANA Cluster. Supplementary Table 3: Substance Use. Supplementary Table 4: Alcohol Consumption.

## Data Availability

Data are not publicly available but can be provided upon reasonable request.
