# Correction to “Brain iron distribution in transdiagnostic mental”

**DOI:** 10.1002/pcn5.70269

**Published:** 2025-12-07

Coray RC, Berberat J, Kagerer SM, Perroud N, Gaviria LJ, Piguet C, et al. Brain iron distribution in transdiagnostic mental health burden. Psychiatry Clin Neurosci Rep. 2025;4:e70243. https://doi.org/10.1002/pcn5.70243

In the published article, the author's name “Lopez Julian Gaviria” and the fourth affiliation “Department of Psychiatry, Vrije Universiteit, Amsterdam, The Netherlands” were incorrect.

The correct author name is “Julian Gaviria Lopez” and the correct affiliation is “Psychiatry Department, Amsterdam UMC, Location Vrije Universiteit Amsterdam, Amsterdam, The Netherlands”.

The online version of this article has been corrected accordingly.

We apologize for this error.

