# Supplementary material for: Brain iron distribution in transdiagnostic mental health burden
Source: PCN Rep. 2025 Nov 19;4(4):e70243. doi: 10.1002/pcn5.70243 (PMC12627966; doi:10.1002/pcn5.70243)
Supplement: Supplementary file 1 — Supplementary Figure 1: Missing data pattern. The missingness appears to follow a systematic structure along the diagonal, suggesting that the data is not Missing Completely At Random (MCAR). Since the missingness is dependent on observed data (e.g., specific brain regions with higher noise levels in imaging), missing data is assumed to be Missing At Random (MAR). Supplementary Figure 2. PCA projection with three clusters. Data points are colored by cluster assignment (Cluster 1 = red, Cluster 2 = green, Cluster 3 = blue). Ellipses represent the 95% confidence interval for each cluster in the two‐dimensional principal component space. Supplementary Table 1: Demographic Table. Supplementary Table 2: Medication per DIANA Cluster. Supplementary Table 3: Substance Use. Supplementary Table 4: Alcohol Consumption. [file PCN5-4-e70243-s001.docx]

**Supplementary Material**


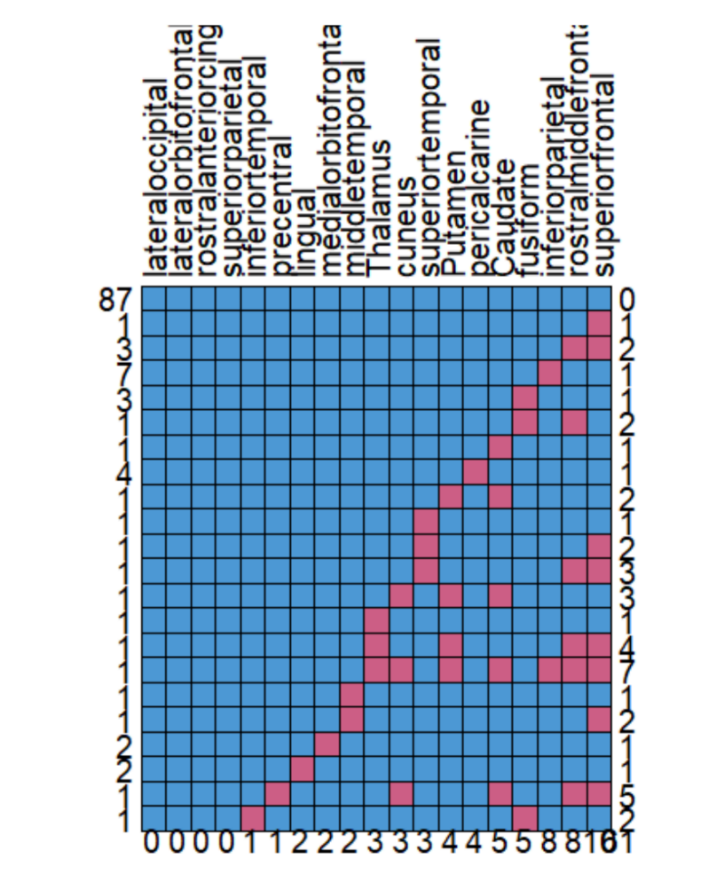


Supplementary Figure 1: **Missing data pattern**. The missingness appears to follow a systematic structure along the diagonal, suggesting that data is not Missing Completely At Random (MCAR). Since the missingness is dependent on observed data (e.g., specific brain regions with higher noise levels in imaging), missing data is assumed to be Missing At Random (MAR).


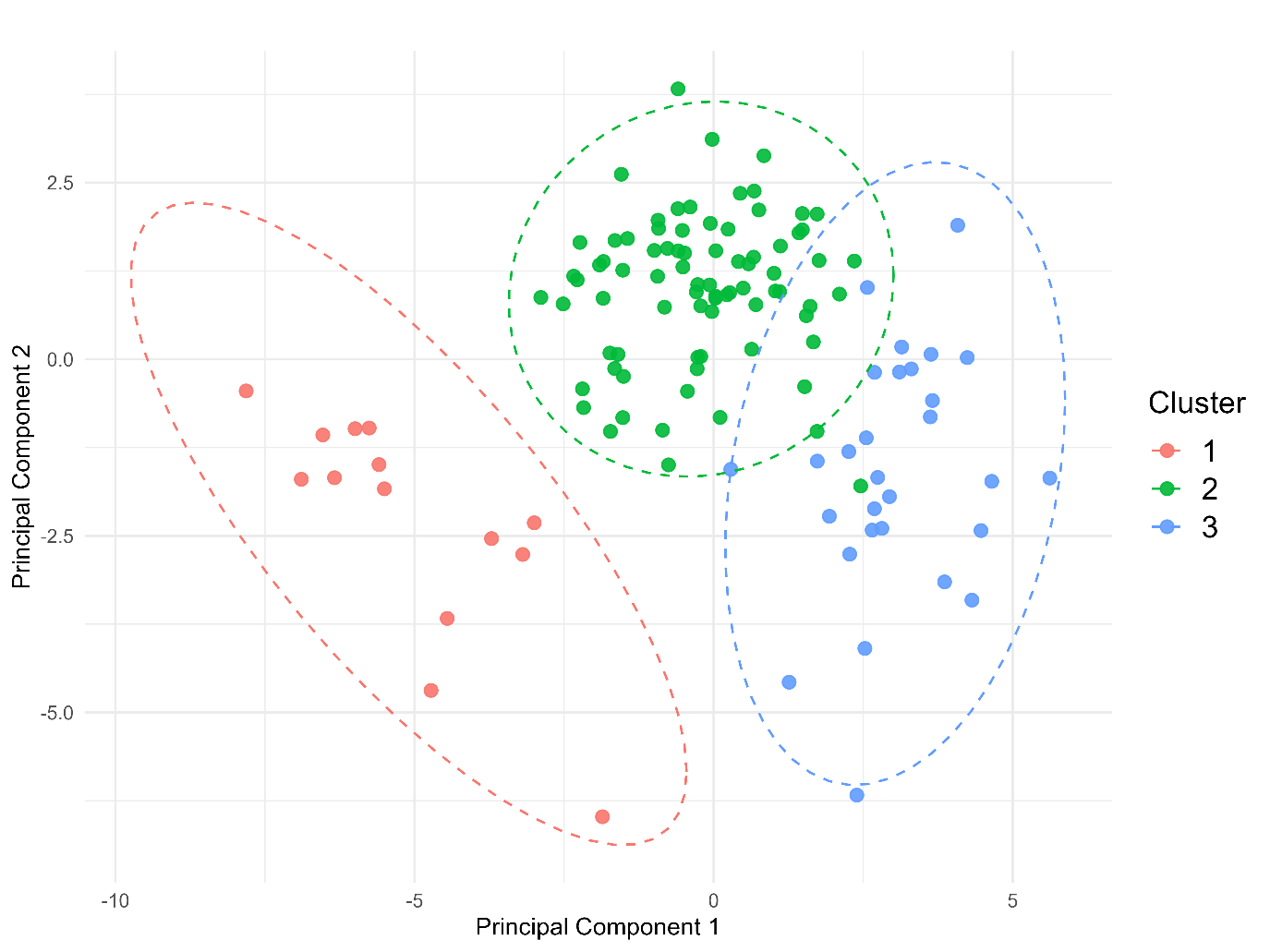


Supplementary Figure 2. **PCA projection with three clusters***. Data points are colored by cluster assignment (Cluster 1 = red, Cluster 2 = green, Cluster 3 = blue). Ellipses represent the 95% confidence interval for each cluster in the two-dimensional principal component space.*

| **Supplementary Table 1: Demographic Table** | | | |
| --- | --- | --- | --- |
|  | **Diana Cluster** | | |
|  | **Cluster 1** | **Cluster 2** | **Cluster 3** |
| Female sex n (%) | 7 (47 %) | 49 (62%) | 17 (59 %) |
| Male sex n (%) | 8 (53 %) | 30 (38%) | 12 (41 %) |
| Mean age (SD), years | 27.5 *(12.1)* | 23 *(6.8)* | 24.6 *(5.8)* |
| Body Mass Index (BMI) | 23.4 | 22.3 | 22.9 |
| Number of BD | 9 | 0 | 0 |
| Number of ADHD | 2 | 8 | 4 |
| Number of BPD | 0 | 10 | 6 |
| Number of patient offspring | 3 | 29 | 9 |
| Number of controls | 0 | 15 | 4 |
| Number of control offspring | 1 | 17 | 6 |

Demographic and clinical characteristics by Diana cluster, including sex distribution, mean age (SD), and number of participants with BD, ADHD, BPD, as well as patient and control group assignments.

| **Supplementary Table 2: Medication per Diana Cluster** | | | |
| --- | --- | --- | --- |
|  | **Active Substance** | **Participants (*N*)** | **Percentage (*%*)** |
| Cluster1 | Methylphenidate | 2 | 14.3 |
|  | Melatonin | 1 | 7.1 |
|  | Lithium | 2 | 14.3 |
|  | Lamotrigine | 2 | 7.1 |
|  | Citalopram | 1 | 7.1 |
|  | Aripiprazole | 2 | 14.3 |
| Cluster2 | Sertraline | 1 | 1.2 |
|  | Salbutamol | 2 | 2.5 |
|  | Methylphenidate | 6 | 7.5 |
|  | Lisdexamfetamine | 1 | 1.3 |
|  | Levothyroxine | 1 | 1.3 |
|  | Fluoxetine | 2 | 2.5 |
|  | Escitalopram | 2 | 2.5 |
|  | Dexmethylphenidate | 3 | 3.8 |
|  | Asenapine | 1 | 1.3 |
|  | Aripiprazole | 1 | 1.3 |
| Cluster3 | Selenium | 1 | 3.4 |
|  | Methylphenidate | 4 | 13.8 |
|  | Dexmethylphenidate | 1 | 3.4 |
|  | Oral Contraceptive | 2 | 6.9 |

Active substances per cluster and absolute number of participants taking each medication. Participants may appear in more than one category if they take multiple active substances. Combinations are therefore included within the counts and not reported separately.

| **Supplementary Table 3: Substance Use** | | | |
| --- | --- | --- | --- |
|  | **History** | **No History** | **Missing Data** |
| Cluster 1 (n=15) | 7 (50%) | 7 (50%) | 1 |
| Custer 2 (n=79) | 11 (15%) | 64 (85%) | 4 |
| Cluster 3 (n=29) | 4 (15%) | 23 (85%) | 2 |

Substance use history per Diana cluster, assessed with the Diagnostic Interview for Genetic Studies (DIGS). Absolute numbers are reported in the table, with percentages calculated within clusters (excluding missing data). A chi-square test indicated a significant association between cluster membership and substance use history, *χ*²(2)=9.98, *p*=.007.

| **Supplementary Table 4: Alcohol Consumption** | | | | | |
| --- | --- | --- | --- | --- | --- |
|  | **No alcohol consumption** | **Abuse** | **Dependence** | **Abuse and dependence** | **Missing Data** |
| Cluster 1 (n=15) | 7 (50%) | 0 (0%) | 5 (36%) | 2 (14%) | 1 |
| Custer 2 (n=79) | 70 (93%) | 0 (0%) | 3 (4%) | 2 (3%) | 4 |
| Cluster 3 (n=29) | 22 (82%) | 4 (15%) | 1 (4%) | 0 (0%) | 2 |

Alcohol consumption patterns per Diana cluster, assessed with the Diagnostic Interview for Genetic Studies (DIGS). Absolute numbers are reported, with corresponding percentages calculated within clusters (excluding missing data). A chi-square test indicated a significant association between cluster membership and alcohol consumption pattern, *χ*²(6)=37.7, *p*<.001.
